# Case report: Treating a co-existence of hidradenitis suppurativa and psoriasis with different therapeutic approaches

**DOI:** 10.12688/f1000research.21216.2

**Published:** 2020-12-22

**Authors:** Eleftheria Tampouratzi, Theodora Kanni, John Katsantonis, Theodora Douvali

**Affiliations:** 1 Tzaneio Hospital, Piraeus/Athens, Greece; 2 Andreas Sygros University Hospital, Athens, 161 21, Greece

**Keywords:** hidradenitis suppurativa, psoriasis, certolizumab, brodalumab

## Abstract

Hidradenitis suppurativa and psoriasis are considered chronic inflammatory diseases suggesting the existence of common pathogenetic pathways. We present two cases of comorbid psoriasis and hidradenitis suppurativa, treated with certolizumab pegol and brodalumab due to failure of response to other conventional therapies. Monoclonal antibody therapies have revolutionized the treatment of chronic inflammatory disorders such as psoriasis and hidradenitis suppurativa. Given the good clinical response to anti-IL-17 and anti-tumor necrosis factor agents in patients undergoing psoriasis and hidradenitis treatment, investigations on this direction could represent the starting point in new therapeutic approach for revolutionary treatment in these difficult-to-treat diseases.

## Introduction

Hidradenitis suppurativa (HS) and psoriasis are considered chronic inflammatory diseases suggesting the existence of common pathogenetic links ^1– 3^. Patients with psoriasis and HS have elevated levels of tumor necrosis factor (TNF) and interleukin-17 (IL-17) in lesional and non lesional tissues, which has been the justification for selective targeting of these inflammatory pathways ^4– 7^. We present two cases of co-existence of psoriasis and HS treated with certolizumab pegol and brodalumab due to the peculiarities of treatment with other therapies.

## Case report

**Figure 1.**
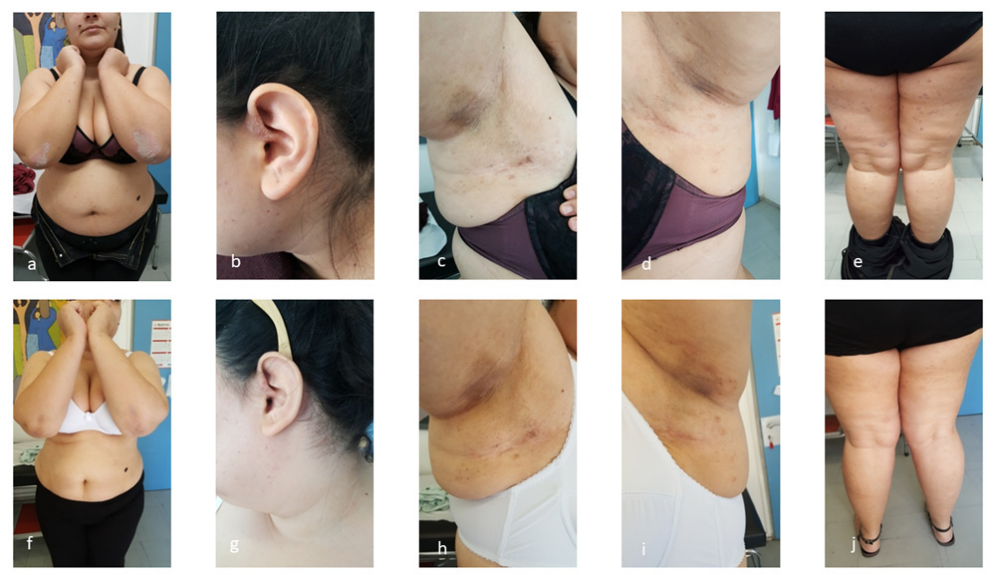
Psoriatic and HS lesions of patient 1. ( **a**– **e**) Psoriatic and HS lesions of first patient before treatment with certolizumab pegol. ( **f**– **j**) Psoriatic and HS lesions of first patient after treatment with certolizumab pegol.

The first patient, a 27-year-old Caucasian woman, presented with extensive psoriasis vulgaris covering her head, trunk, lower limbs over a period of 5 years, with a recent PASI (Psoriasis Area Severity Index) score of 10.5 and 10% BSA (Body Surface Area) score. She, also, suffered from psoriatic arthritis with axial joint involvement (manifestations of hierolagonitis) over the previous 2 years and moderate HS of Hurley II stage disease on the axillae over the last year, with IHS4 (International Hidradenitis Suppurativa Severity Scoring System) score 10 ( Figure 1a, b, c, d, e). Despite the limited extent of the lesions, the patient presented considerable pain, discomfort and substantial negative effect on quality of life. Patient’s DLQI (Dermatology Life Quality Index) score was, also very high, 21. The patient didn’t have a positive family history for the above diseases and the molecular control for HLA-B27 was negative. Previous treatments with topical corticosteroids and methotrexate for one year were not effective and treatment with apremilast for 8 months didn’t offer clinical improvement in both diseases. The patient underwent comprehensive laboratory investigations, including complete blood cell count, chemistry panel, tuberculosis (Quantiferon-TB Gold test), human immunodeficiency virus and hepatitis B and C screening and chest x-ray. Since all these examinations revealed values within normal limits and because of the patient’s desire for childbirth, she was treated with certolizumab pegol (CZP). The initial dose was 400mg, followed by 400mg every 2 weeks. Treatment with CZP significantly improved psoriasis and psoriatic arthritis at week 8 and HS at week 12. The PASI score after treatment was 1, BSA was 2%, whereas IHS4 score was 1. Except the clinical improvement, the DLQI score was impressively reduced to 2. ( Figure 1f–i). She continues treatment 9 months after and at 3 months follow-up is fully controlled.

**Figure 2.**
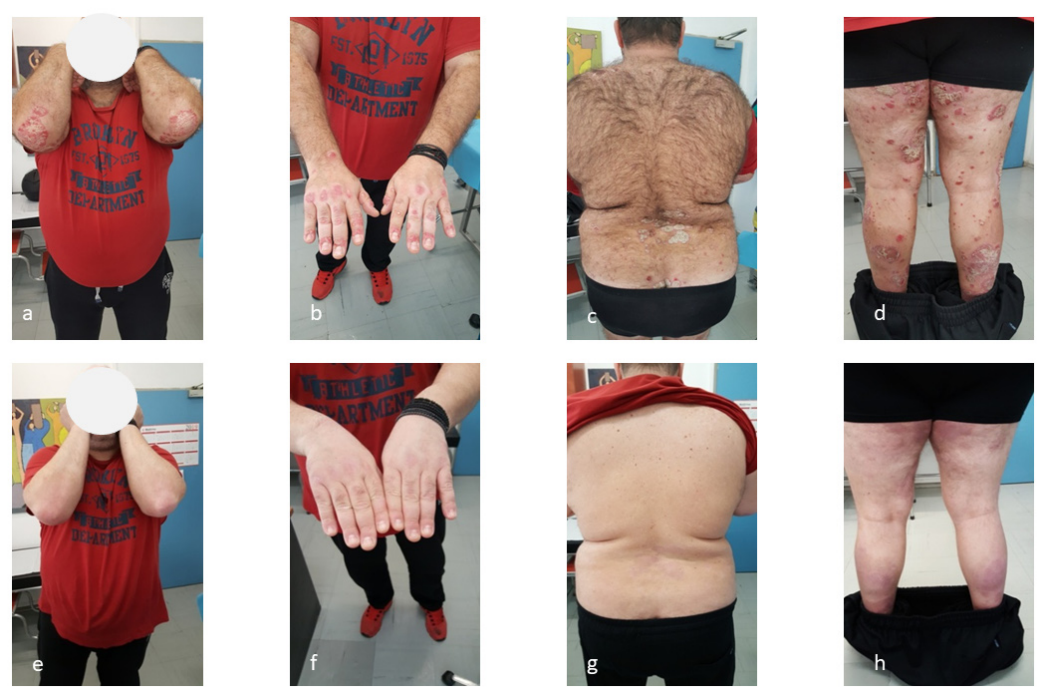
Psoriatic lesions of patient 2. ( **a**– **d**) Psoriatic lesions of second patient before treatment with brodalumab. ( **e**– **h**) Psoriatic lesions of second patient after treatment with brodalumab.

The second patient, a 42-year-old Caucasian man, was referred to our hospital’s dermatological department with multiple, itchy, scaly, red-gray psoriatic plaques covering almost all his body: scalp, arms, trunk, thighs ( Figure 2a–d) for the previous 6 months, over a history of 10 years psoriatic disease (recent PASI: 18.5, BSA: 45%). The patient, also, experienced concomitant psoriatic arthritis with peripheral joint involvement and dactylitis discomfort over the previous 10 years, and moderate HS of Hurley II stage disease appearing on the groin area in the previous year. The IHS4 score was 10. The above diseases had a negative impact factor on his quality of life with DLQI 25. The patient’s family history was positive: his mother and sister were also suffering from psoriasis. The patient had until recently received almost all the available therapies related to his diseases: cyclosporine for 2 years interrupted due to urea and creatinin increase (examinations restored after discontinuation), methotrexate and golimumab for 3 years with improvement only in psoriatic arthritis, adalimumab ustekinumab and secukinumab, with a partial response. After a complete laboratory examination, with results in normal limits, the patient started therapy with brodalumab. The initial dose was 210 mg at weeks 0, 1, 2 followed by 210 mg every 2 weeks. His psoriasis and psoriatic arthritis were highly improved at week 8 ( Figure 2 e–h), as was HS at week 16. The PASI score after treatment was 1.5, the BSA was 8%, while IHS4 score was reduced to 3. He has continued treatment for 1 year; at 3 months follow-up he reported improvement in his quality of life and the DLQI score was 1.

## Discussion

Monoclonal antibody therapies have revolutionized the treatment of chronic inflammatory disorders such as psoriasis and HS. CZP is a TNF inhibitor that does not have a fragment crystallizable (Fc) region, which is normally present in a complete antibody and therefore it does not cause antibody-dependent cell-mediated cytotoxicity ^8– 10^. In contrast to other whole-antibody anti-TNFs, CZP crosses the placenta only by passive diffusion and could therefore be considered as the first-line choice of treatment for women who wish to become pregnant. Since CZP is an anti-TNF drug, therapies which have good clinical response in both psoriasis/psoriatic arthritis and HS, it was chosen as the treatment of choice in our case since it also has a safe profile for possible future pregnancy.

Brodalumab is a monoclonal antibody against human IL-17 receptor A (IL-17RA). Given its efficacy in psoriasis and its mechanism of action in psoriatic arthritis and HS, due to the patient’s non response to all the available treatment options it was decided its use on the above combination diseases ^11– 14^.

It is well known that psoriasis and HS likely share immunopathogenetic pathways, including involvement of IL-17 and TNF. Given the good clinical response to anti-IL 17 and anti-TNF drugs in psoriasis and HS treatment, investigations into this direction could represent a starting point for a new therapeutic approach for revolutionary treatment of two difficult to treat diseases.

## Data availability

All data underlying the results are available as part of the article and no additional source data are required.

## Consent

Written informed consent for publication of their clinical details and clinical images was obtained from the patients.
