# Supplementary material for: Case report: Treating a co-existence of hidradenitis suppurativa and psoriasis with different therapeutic approaches
Source: F1000Res. 2020 Dec 22;8:2002. Originally published 2019 Nov 26. [Version 2] doi: 10.12688/f1000research.21216.2 (PMC7791350; doi:10.12688/f1000research.21216.2)
Supplement: Peer review history

# Peer review: Reviewer response for version 2

**DOI:** 10.5256/f1000research.30681.r76345

**Published:** 2021-01-08

**Authors:** Kevin K. Wu

**Affiliations:** 1 University of California, Irvine, Irvine, CA, USA

Accept. No additional comments.

Are enough details provided of any physical examination and diagnostic tests, treatment given and outcomes?

No

Is the background of the cases’ history and progression described in sufficient detail?

Yes

Is sufficient discussion included of the importance of the findings and their relevance to future understanding of disease processes, diagnosis or treatment?

Yes

Is the conclusion balanced and justified on the basis of the findings?

Yes

Reviewer Expertise:

NA

I confirm that I have read this submission and believe that I have an appropriate level of expertise to confirm that it is of an acceptable scientific standard.

# Peer review: Reviewer response for version 2

**DOI:** 10.5256/f1000research.30681.r76343

**Published:** 2021-01-07

**Authors:** Georgios Nikolakis

**Affiliations:** 1 Department of Dermatology, Venereology, Allergology and Immunology, Dessau Medical Center, Brandenburg Medical School Theodor Fontane, Dessau, Germany; 2 European Hidradenitis Suppurativa Foundation, Dessau, Germany

In this version of the manuscript Tampouratzi and colleagues have provided all validated scores and patient reported outcomes to strengthen the quality of their manuscript. I would like to congratulate them and have no further comments.

Are enough details provided of any physical examination and diagnostic tests, treatment given and outcomes?

No

Is the background of the cases’ history and progression described in sufficient detail?

Partly

Is sufficient discussion included of the importance of the findings and their relevance to future understanding of disease processes, diagnosis or treatment?

Partly

Is the conclusion balanced and justified on the basis of the findings?

No

Reviewer Expertise:

HS, sebocytes, acne, melanoma, allergy

I confirm that I have read this submission and believe that I have an appropriate level of expertise to confirm that it is of an acceptable scientific standard.

# Peer review: Reviewer response for version 1

**DOI:** 10.5256/f1000research.23356.r71707

**Published:** 2020-09-28

**Authors:** Kevin K. Wu

**Affiliations:** 1 University of California, Irvine, Irvine, CA, USA

Thank you for these two interesting cases. One major thing missing from this paper are objective measurements of improvement after starting the respective therapies. Simply stating "His psoriasis and psoriatic arthritis were highly improved at week 8 ( Figure 2 e–h), as was HS at week 16" or "Treatment with CZP significantly improved psoriasis and psoriatic arthritis at week 8 and HS at week 12 ( Figure 1f–i)." does not give the reader an objective measurement of how much better the patient's disease process became following therapy. Did you measure PASI/IGA scores? Did the patients improve based on their Hurley or HISCR scores? This paper should only be accepted for indexing after including some essential, objective outcome measures. If these measures cannot be obtained, then this manuscript should be rejected for indexing.

Are enough details provided of any physical examination and diagnostic tests, treatment given and outcomes?

No

Is the background of the cases’ history and progression described in sufficient detail?

Yes

Is sufficient discussion included of the importance of the findings and their relevance to future understanding of disease processes, diagnosis or treatment?

Yes

Is the conclusion balanced and justified on the basis of the findings?

Yes

Reviewer Expertise:

Psoriasis, HS, atopic dermatitis, biologics, epidemiology.

I confirm that I have read this submission and believe that I have an appropriate level of expertise to confirm that it is of an acceptable scientific standard, however I have significant reservations, as outlined above.

# Response

**Published:** 2020-12-08

**Authors:** Theodora Kanni

**Affiliations:** Andreas Sygros University Hospital, Greece

Dear Dr. Wu,

 We revised our manuscript according to your comments.

 We provide severity assessment before and after treatment for both cases.

 The clinical improvement for psoriasis and hidradenitis is estimated using the PASI (Psoriasis Area Severity Index) score, BSA (Body Surface Area), and IHS4 (International Hidradenitis Suppurativa Severity Scoring System) score, while the impact on the quality of life is estimated with the DLQI (Dermatology Life Quality Index) score.

# Peer review: Reviewer response for version 1

**DOI:** 10.5256/f1000research.23356.r57162

**Published:** 2019-12-11

**Authors:** Georgios Nikolakis

**Affiliations:** 1 Department of Dermatology, Venereology, Allergology and Immunology, Dessau Medical Center, Brandenburg Medical School Theodor Fontane, Dessau, Germany; 2 European Hidradenitis Suppurativa Foundation, Dessau, Germany

I have prepared a PDF file with most of the points I think need to be addressed in order to make this case acceptable for indexing - please find the file here *. *For the second case we have really no proof, even a single photo, showing that brodalumab led to improvement of HS. Both Psoriasis and HS need to be assessed using both descriptive terms but also objective and validated scoring systems, to quantify the improvement.

 Moreover, the improvement of HS under certolizumab pegol is not clear for me, since I cannot tell that inflammatory lesions (nodules, abscesses or sinus tracts) have decreased after therapy.

 Since I believe that this case report can add to the current literature, opening ways for more anti-inflammatory treatments for HS, I think that updating the documentation accordingly and providing some proof for the improvement of HS will make the manuscript acceptable for indexing.

Are enough details provided of any physical examination and diagnostic tests, treatment given and outcomes?

No

Is the background of the cases’ history and progression described in sufficient detail?

Partly

Is sufficient discussion included of the importance of the findings and their relevance to future understanding of disease processes, diagnosis or treatment?

Partly

Is the conclusion balanced and justified on the basis of the findings?

No

Reviewer Expertise:

HS, sebocytes, acne, melanoma, allergy

I confirm that I have read this submission and believe that I have an appropriate level of expertise to state that I do not consider it to be of an acceptable scientific standard, for reasons outlined above.

# Response

**Published:** 2020-12-08

**Authors:** Theodora Kanni

**Affiliations:** Andreas Sygros University Hospital, Greece

Dear Dr. Nikolakis,

 We revised our manuscript according to your comments.

 We provide severity assessment before and after treatment for both cases.

 The clinical improvement for psoriasis and hidradenitis is estimated using the PASI (Psoriasis Area Severity Index) score, BSA (Body Surface Area), and IHS4 (International Hidradenitis Suppurativa Severity Scoring System) score, while the impact on the quality of life is estimated with the DLQI (Dermatology Life Quality Index) score.

 Regarding your comment about the photographic documentation of HS improvement of the second patient, the patient denied taking photos. The location of his HS lesions is on the groin area and we did not have the patient’s consent for photographic documentation.

# Peer review: Reviewer response for version 1

**DOI:** 10.5256/f1000research.23356.r57163

**Published:** 2019-12-10

**Authors:** Thrasyvoulos Tzellos

**Affiliations:** 1 Department of Clinical Medicine, University of Tromsø, Tromsø, Norway

I suggest changing the title to:

 "Treating co-existence of hidradenitis suppurativa and psoriasis"

 Introduction:

- Please change "We present two cases of comorbidity of psoriasis and HS” to ”We present two cases of co-existence of psoriasis and HS”.

For case report 1:

- Please provide a severity assessment for psoriasis.
- The authors only refer to “extensive”. It would be important to report PASI or another measure of severity assessment. 

For case report 2:

- Please provide a severity assessment for psoriasis as for case 1.
- Also it reported negative impact on quality of life. Please provide a measure if available. For example VAS pain 6 or DLQI 10.

Are enough details provided of any physical examination and diagnostic tests, treatment given and outcomes?

Yes

Is the background of the cases’ history and progression described in sufficient detail?

Partly

Is sufficient discussion included of the importance of the findings and their relevance to future understanding of disease processes, diagnosis or treatment?

Yes

Is the conclusion balanced and justified on the basis of the findings?

Yes

Reviewer Expertise:

Hidradenitis suppurativa, atopic dermatitis, biologics, Evidence based medicine

I confirm that I have read this submission and believe that I have an appropriate level of expertise to confirm that it is of an acceptable scientific standard.

# Response

**Published:** 2020-12-08

**Authors:** Theodora Kanni

**Affiliations:** Andreas Sygros University Hospital, Greece

Dear Prof. Tzellos,

 We revised our manuscript according to your comments. All your comments were taken into account and please find below the answers:

- We changed the title according to your suggestion.
- In the introduction section, we changed the term comorbidity with the term co-existence.
- Finally, we provide severity assessment before and after treatment for both psoriasis and hidradenitis, as well as DLQI score for the impact on the quality of life.
